# A nurse-led up-titration clinic improves chronic heart failure optimization of beta-adrenergic receptor blocking therapy - a randomized controlled trial

**DOI:** 10.1186/1756-0500-7-668

**Published:** 2014-09-23

**Authors:** Andrea Driscoll, Piyush Srivastava, Deidre Toia, Jackie Gibcus, David L Hare

**Affiliations:** School of Nursing and Midwifery, Deakin University, 225 Burwood Highway, Melbourne, Burwood, Australia; Department of Cardiology & Department of Medicine, University of Melbourne, Austin Health, Burgundy St Heidelberg, 3081 Melbourne, Australia; Department of Cardiology, Austin Health, Burgundy St Heidelberg, 3081 Melbourne, Australia; Cardiovascular Research, University of Melbourne, Burgundy St Heidelberg, 3081 Melbourne, Australia

**Keywords:** Nurse-led clinic, Beta-adrenergic blockers, Heart failure

## Abstract

**Background:**

Beta-adrenergic blockade has been shown to improve left ventricular function, reduce hospital admissions and improve survival in chronic heart failure with reduced ejection fraction (HFrEF), with mortality reduction starting early after beta-adrenergic receptor blocker initiation and being dose-related. The aim of this pilot study was to determine the effectiveness of a nurse-led titration clinic in improving the time required for patients to reach optimal doses of the beta-adrenergic receptor blocking agents.

**Method:**

We conducted a prospective pilot randomized controlled trial. Twenty eight patients with CHF were randomized to optimisation of beta-adrenergic receptor blocker therapy over six months by either a nurse-led titration (NLT) clinic, led by a nurse specialist with the support of a cardiologist in a CHF clinic, or by their primary care physician (usual care (UC)). The primary endpoint was time to maximal beta-adrenergic receptor blocker dose. The secondary end-point was the proportion of patients reaching the target dose of beta-adrenergic receptor blocker by six months.

**Results:**

The patients were predominantly men (72%), age 67 ± 16 years; New York Heart Association (NYHA) functional class I (32%), II (44%) and III (20%); baseline left ventricular ejection fraction 33 ± 10%, and a low mean Charlson co-morbidity score of 2.5 ± 1.4. The time to maximum dose was shorter in the NLT group compared to the UC group (90 ± 14 vs 166 ± 8 days, p < 0.0005). At six months, in the NLT group there were nine patients (82%) on high dose and one patient (9%) on low dose beta-adrenergic receptor blocker compared to the UC group with five (42%) patients reaching maximum dose and five (42%) patients on low dose (p = 0.04). The patients allocated to the NLT group also had significantly less worsening of depression between baseline and six months (p = 0.006).

**Conclusion:**

A NLT clinic improves optimisation of beta-adrenergic receptor blocker therapy through increasing the proportion of patients reaching maximal dose and facilitating rapid up-titration of beta-adrenergic receptor blocker agents in patients with chronic HFrEF.

**Trial registration:**

Australian Clinical Trials Registry (ACTRN012606000383561).

## Introduction

Chronic heart failure with reduced ejection fraction (HFrEF) is a syndrome with high mortality and morbidity [1]. The global burden of chronic heart failure will continue to grow as its incidence approaches 10 per 1000 population [2, 3] with one third of individuals aged 55 years or older predicted to develop the syndrome in their remaining lifetime [4]. As such, despite advances in medical treatment, chronic HFrEF continues to be the most frequent cause of hospitalisation in patients aged 65 years or older [5].

Several pharmacological treatments for chronic HFrEF have resulted in a reduction in hospital admissions, and improvement in both survival and quality of life. The overall efficacy of beta-adrenergic blockade using “beta-adrenergic receptor blockers” has been proven through several large trials [6–10] with international and national expert guidelines stipulating the use of these agents for patients with chronic HFrEF and left ventricular dysfunction [11–13].

## Background

Even in the rigorous clinical trial setting, there has been difficulty getting patients to the optimal doses of beta-adrenergic receptor blocking agents. The percentage of patients reaching the trial specified target dose was 80% in the US Carvedilol Program [6], 64% in the MERIT HF [8, 9] study and 40% in the CIBIS-II trial [7]. This is even more difficult in usual clinical practice [14]. Despite the evidence for the marked benefits of beta-adrenergic blocking agents on patient outcomes in chronic HFrEF, they continue to be under-utilised, both under-prescribed and under-dosed. For heart failure patients in a primary care setting, only 12% had been prescibed beta-adrenergic receptor blocking agents at all, let alone optimal doses, and their use declined with each increase in decade of life [14]. Subsequent chronic HFrEF studies have confirmed that the doses of beta-adrenergic blockers applied in clinical practice are substantially less than the doses achieved in randomized clinical trials and recommended in national guidelines [15–17].

Barriers experienced by primary care physicians in managing chronic HFrEF patients and under-utilization of beta-adrenergic receptor blocking agents include a lack of experience with both initiation and up-titration in the community setting, and also perceptions of side-effects from and contra-indications to beta-adrenergic receptor blocking agents [18].

In clinical practice the cardiologist usually initiates the beta-adrenergic receptor blocker and refers the patient to their primary care physician for up-titration of the doses to the recommended target levels. In practice the later rarely seems to happen. In light of the poor uptake of expert guidelines and reluctance of primary care physicians to up-titrate beta-adrenergic receptor blockers in patients with chronic HFrEF, new strategies are required to fill this “treatment gap”. Because of the high mortality even within the first 12 months after HFrEF diagnosis, it is of paramount importance not to delay the attainment of efficacious doses. In busy cardiology practice, there is rarely the time available to regularly review the patients every couple of weeks, primarily for medication up-titration. The implementation of a nurse-led titration (NLT) clinic for HFrEF patients is one possible strategy. The NLT clinic could optimise the utilization of beta-adrenergic receptor blockers, thereby potentially improving patient morbidity, mortality and the need for hospitalization. A previous randomized control study reported the success of nurse-led heart failure clinics [19]. However, they did not specifically investigate the up-titration of beta-adrenergic receptor blockers. Observational studies have reported an improvement in beta-adrenergic receptor blocking agents in nurse-led heart failure clinics [20] and in community settings [21]. This pilot study fills the gap in the current literature as it is a randomized control trial specifically investigating the titration of beta-adrenergic receptor blocking agents in a nurse-led heart failure clinic.

The aim of this pilot study was to determine the effectiveness of a NLT clinic in the rapid uptitration of beta-adrenergic receptor blocking agents that have been demonstrated to have efficacy in HFrEF. The study was approved by the Human Research Ethics Committee of Austin Health in Melbourne, Australia and was registered with the Australian Clinical Trials Registry (ACTRN012606000383561).

## Methods

### Study population

The study population constituted a prospective cohort of chronic HFrEF patients attending a specialist outpatient heart failure clinic. The clinic operated as a secondary and tertiary referral service for long-term management of complex heart failure patients. All patients received written study information and gave signed, informed consent prior to study entry.

### Patient recruitment

Entry criteria:

- Stable chronic HFrEF patients with current beta-adrenergic receptor blocker therapy at less than half the recommended target dose for the specific agent and requiring up titration. The required entry doses were carvedilol < 12.5 mg twice daily, metoprolol XL < 90 mg daily or bisoprolol < 5 mg daily.
- Impaired left ventricular (LV) systolic dysfunction as documented by gated blood pool scanning or echocardiography. For beta–adrenergic receptor blocker naive patients, the LV assessment had to be within six months of trial entry. For patients already started on low-dose beta-blockade, repeat LV assessment had to be within two months of trial entry.

Exclusion criteria:

- Previously failed an increase in titration of beta-adrenergic receptor blockers in the previous six months. Assessed by a heart failure cardiologist as being medically inappropriate for up titration of beta-adrenergic receptor blocking agents in a NLT clinic. This was predominantly because of the need for tighter cardiology monitoring, necessitating early cardiology review and management changes.
- Unable to read and speak English.

### Study design

The pilot study was a prospective, randomised controlled trial. After the collection of baseline data, patients were allocated to either the NLT or UC group according to computer generated random numbers held in opaque, sealed envelopes by a third party. The number in each group was balanced after every six patients, using the block method.

**Figure 1:**
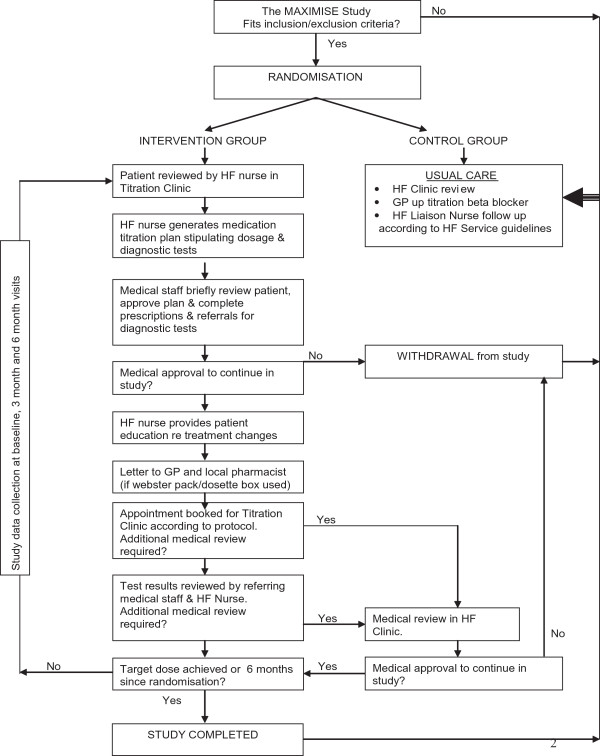
**Study flowchart.**

Patients in both groups had scheduled clinic visits with a heart failure cardiologist at three and six months after randomisation. Whilst patients in the NLT group had regular scheduled visits to the NLT clinic as determined by the nurse, patients in the UC group were contacted monthly by telephone. The study flowchart is described in Figure 1.

The target daily doses were carvedilol 50 mg, metoprolol XL 190 mg and bisoprolol 10 mg. To compare the equivalent doses of different beta-adrenergic receptor blocking agents reached in the two arms of the study, equivalent beta-adrenergic receptor blocker doses were calculated as being approximately equivalent to carvedilol. These were obtained by dividing the metoprolol dose by four and multiplying the bisoprolol dose by five. For example, a 7.5 mg daily dose of bisporolol would be given a carvedilol equivalent total daily dose of 37.5 mg. Low, medium and high daily doses of carvedilol were defined: low dose: ≤12.5 mg, medium dose: >12.5 but ≤25 mg, and high dose: >25 mg up to 50 mg (25 mg twice daily).

### Usual care group (UC)

Patients randomised to the UC group, underwent assessment by a cardiologist at the heart failure clinic. Management recommendations were made. Information describing beta-adrenergic receptor blocker up-titration was communicated in writing to both the patient and their primary care physician. If patients were receiving home visits by heart failure nurses then these continued. The patients were not reviewed again in the heart failure clinic until their scheduled cardiologist visits at both three and six months post randomisation.

### Study intervention (Nurse-led titration (NLT))

Patients in the intervention group were reviewed by the heart failure nurse in the clinic weekly, fortnightly or monthly until they reached the maximum optimal dose of beta-adrenergic receptor blocking agents and had attended for the six month intervention period. At each visit the heart failure nurse undertook a clinical examination of the patient, determined appropriate medication changes, tests and referrals, and educated the patient concerning medication changes. The referring Cardiologist also reviewed the patient and approved proposed changes, completed medication prescriptions and referral forms. Each patient received a printed list of current medications including the new titrated dose of medications. It is important to note that, whilst the titration clinic was run by the heart failure nurse, a cardiologist was available to briefly see each patient and, especially in patients who had significant co-morbidities and up-titration difficulties, guide the nurse in the up-titration process.

### Study endpoints and data collection

The primary endpoint was the difference in time taken to reach the optimal tolerated dose of beta–adrenergic receptor blocker as determined in consultation between the heart failure nurse and Cardiologist. Secondary end-point was the likelihood of reaching maximal dose of beta-adrenergic receptor blocking agents by six months. Tertiary end-points of interest were all-cause and heart failure hospital admissions, all-cause and heart failure emergency department attendances, changes in general quality of life - as measured on the Minnesotta Living with Heart Failure (MLWHF) questionnaire [22] and changes in depressed mood - as measured using the Cardiac Depression Scale (CDS) score [23].

Baseline demographic and medical history data were collected during their first visit prior to randomisation. The complexity of the patients was rated using the Charlson Co-morbidity Index [24]. All patients were followed up for six months. Data concerning medication adherence and adverse events were recorded at each visit. At three and six months, the current dose of beta-adrenergic receptor blocker, the dates of up-titration, mortality, emergency department attendances, and the number of hospital admissions were recorded. The MLWHF and CDS questionnaires were completed at baseline, three months and at the end of the study, higher scores indicating worse quality of life and depression respectively.

### Statistical analysis

Sample size calculation was based on the primary outcome of time to maximum dose. A sample size of 121 patients in each group would have 80% power to detect a significant variance of 10% in the primary endpoint of time to maximum dose during six month follow up assuming a two sided α of 0.05. However, the pilot study was stopped prematurely due to financial constraints and slower than expected recruitment rates.

SPSS for Windows version 21 (SPSS Inc 2012) was used to analyse the data. All missing values were excluded from the analysis. Chi-square analysis was used for discrete variables (with calculation of odds ratio and 95% confidence intervals where appropriate). Students t-test was used for normally distributed variables. A value of *P* < 0.05 was considered statistically significant.

A Kaplan-Meier curve with a log likelihood ratio was used to compare our primary endpoint of time to maximum dose of beta-adrenergic blocking agents between NLT clinic and UC groups.

## Results

**Figure 2:**
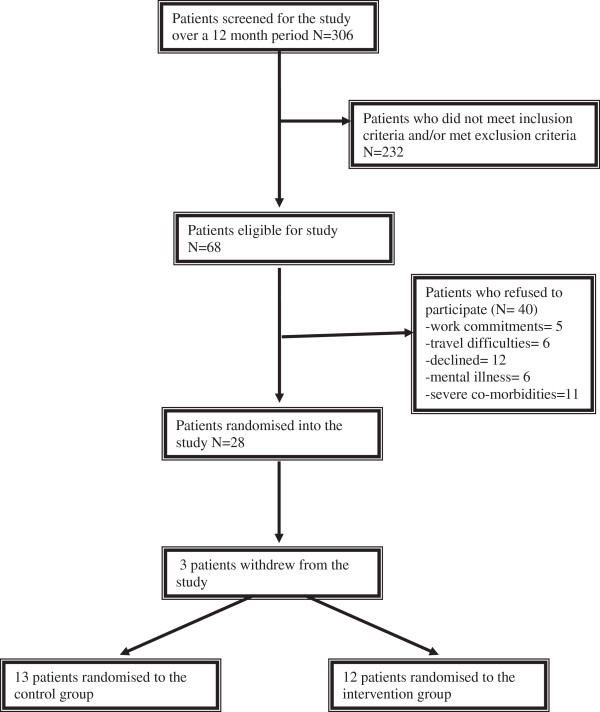
**Flowchart of patient enrolment.**

During the 12 month recruitment period for the pilot study, 306 patients were screened (Figure 2). Of 68 eligible patients, 40 refused participation and three subsequently withdrew, leaving 25 patients in the randomised study. Of the three that withdrew, two patients were from the intervention arm: one was too ill to continue so he decided to withdraw and one was unable to attend clinic due to work commitments. One patient withdrew from the usual care arm as he was travelling for six months and would not be contactable.

Baseline characteristics of patients recruited into the pilot study are presented in Table 1. The majority of the cohort was male (72%), aged over 65 years and had multiple co-morbidities with a mean Charlson Co-morbidity Index of 2.5 ± 1.4. There were no significant differences between the two groups in baseline characteristics.

**Table 1:** **Baseline characteristics of patients enrolled in NLT group compared to UC group**

Patient characteristic	Nurse-led titration group (NLT)	Usual care group (UC)	P value
	Number of patients	Number of patients	
	(n = 12)	(n = 13)	
*Demographic characteristics*			
Age	65 ± 14.2	68 ± 18.7	0.7
Male	9	9	0.6
Living alone	4	4	0.6
Caucasian	7	10	0.5
*Co-morbidities*			
Mean Charlson Co-morbidity Index score (±SD)	2.7 ± 2	2.4 ± 1.3	0.7
Chronic obstructive pulmonary disease	5	2	0.2
Diabetes mellitus	3	4	0.5
Chronic renal impairment	2	3	1.0
Hypertension	3	8	0.07
Dyslipidaemia	3	5	0.4
Atrial fibrillation	1	5	0.2
Ischaemic heart disease	4	3	0.5
*Heart failure profile*			
NYHA class I/II/III/IV	17/3/1	7/4/2/0	0.02
LVEF( ±SD)	34% ± 9%	31% ± 11%	0.5
*Pharmacotherapy at baseline*			
Beta-blocking agents			
ACEIs	11	10	0.6
ARBs	0	4	0.09
Spironolactone	6	4	0.3
Loop diuretic	7	4	0.2
Digoxin	1	1	0.9
Nitrates	3	2	0.9
Aspirin	4	8	0.2
Warfarin	2	1	0.5
Amiodarone	1	2	0.6
Statin	3	4	0.5
*Quality of life*			
MLWHF (±SD)	33 ± 20	37.6 ± 28	0.7
CDS (±SD)	90 ± 27	77 ± 27	0.3

NYHA = New York Heart Association.

LVEF = left ventricular ejection fraction.

ACEI = angiotensin converting enzyme inhibitor.

ARB = angiotensin receptor blocker.

MLWHF = Minnesota Living With Heart Failure.

CDS = Cardiac Depression Scale.

### Time to maximum dose

**Figure 3:**
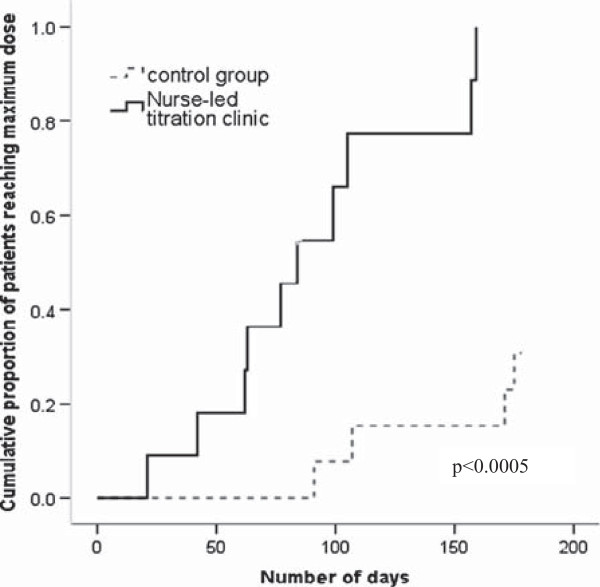
**Cumulative proportion of patients reaching maximum dose of beta-adenergic blockers over six months.**

The NLT group significantly improved time to maximal dose and increased the proportion of patients reaching maximal dose. On average, patients in the NLT group significantly reached optimal dose of beta-adrenergic receptor blocker in nearly half the time compared to those patients in UC group (mean:90 ± 14 days, 95% CI 63.15-118.5 day versus 166 ± 8 days, 95% CI 150.67-182.26 days, p < 0.0005 respectively) (Figure 3). At six months, there was a significantly higher proportion of patients reaching maximal dose of beta-adrenergic receptor blockers in the NLT group compared to UC (10, 91% versus 4, 31%, p = 0.001 respectively). Power calculations were recomputed based on the smaller sample size and larger size of the effect. At a significance level of 0.05 and variance of 55% we had 80% power to detect a significance difference in our primary and secondary endpoints.

### Dosage of beta–adrenergic receptor -adrenergic receptor blockers at 3 and 6 months

At three months, six (50%) patients in the NLT group had their beta-adrenergic receptor blocker optimised to high dose compared to two (15%) in the UC group (p = 0.008) (Table 2). At six months, in the NLT group there were nine (82%) patients on high dose compared with no patients on low dose beta-adrenergic receptor blocker (p = 0.04). Only one patient required a dose reduction due to hypotension and bradycardia. Five (42%) patients in the UC group reached maximum dose at six months (p = 0.04). There were no adverse events associated specifically with NLT.

**Table 2:** **Dose of beta–adrenergic receptor -adrenergic receptor blockers at three and six months**

Beta–adrenergic receptor -adrenergic receptor blockers prescribed	Nurse-led titration group (NLT) (n = 11)	Usual care group (UC) (n = 13)	P value
*At 3 months*			
Low dose	1	8	0.008
Medium dose	5	3	
High dose	6	2	
*At six months*			
Low dose	1	5	0.04
Medium dose	1	2	
High dose	9	5	

### Hospital admissions and emergency department attendances

Overall six (25%) patients experienced a hospital admission and/or presentation to the emergency department with no significant difference between groups (p = 0.1). There was one (9%) hospitalisation (no emergency department presentations) in the NLT group, due to peripheral vascular disease. There were five (39%) hospitalisations/emergency department presentations in the UC group, one patient for exacerbation of HFrEF, the others for prostatectomy because of benign prostatic hyperplasia, a urinary tract infection, an elective admission for an electrophysiology study and a final one for whom the cause could not be ascertained as access to medical records was unavailable. Overall, one patient (in the NLT group) died during the pilot study, this being due to septicaemia after a toe amputation.

### Quality of life scores

On the MLWHF, there was no significant difference between the groups at any time point, although there was a slight trend towards the intervention group having slightly less deterioration over the six months in overall quality of life than the UC control group (mean difference +6.7 ± 16.2 vs +9.5 ± 10.8; p = 0.6).

Mean CDS scores were higher in the NLT group (90 ± 27) at baseline compared to the UC group (77 ± 27) indicating more depressive symptoms in these patients prior to randomisation. However, in the NLT group depression did not continue to worsen as it did in the UC control group, the mean differences in CDS from baseline to six months being -1.8 ± 11.98 vs 17.85 ± 18.44 respectively (p = 0.006).

## Discussion

This pilot study has shown that, in selected patients with cardiologist support, a NLT clinic facilitates rapid uptitration to maximum beta-adrenergic receptor blocking agent doses in patients with HFrEF. In addition, by six months there were a greater proportion of patients who had reached optimal doses. This is an important finding, given that the mortality benefits of beta-adrenergic receptor blockers are evident within the first few months of treatment.

The nurses involved in the up-titration clinic were experienced heart failure nurses. Even though a cardiologist was available to discuss all the patients, the cardiologist time required was generally only a couple of minutes, rather than up to thirty minutes per patient, the latter generally being the time taken to consult with these complex patients. There were no adverse events specifically related to NLT.

Patients in both groups were still allowed to have home visits by heart failure nurses. However, these domiciliary heart failure nurses were only allowed to encourage beta-adrenergic receptor blocker up-titration through the primary care physicians. Both patients and the primary care physicians in the UC group were given written information on the up-titration of beta-adrenergic receptor blocking agents. In spite of this, only 15% of this group of patients reached maximal target doses by three months after randomisation. Thus information and precise recommendations to primary care physicians seems an inadequate strategy for optimal use of beta-adrenergic receptor blocking agents. All of the UC group patients were seen routinely by a specialist heart failure cardiologist at three months. This could explain why there was a further increase in the number of UC patients (42%) reaching the maximal doses at six months. Whilst there were trends for improved quality of life, the study was under-powered for this. Significantly there was an improvement in depression at six months with patients in the NLT group having a reduction in CDS score (-1.8 ± 11.98) compared to UC (17.85 ± 18.44) (p = 0.006).

The pilot study was undertaken with a smaller number of subjects than originally planned. The power calculations were done assuming an effect size of only 10%. However, because of the much greater than expected effect of the intervention, the benefits were highly significant.

Previous studies of up-titration of heart failure medications have been only observational in design. One non-randomised, observational study from the United Kingdom found the optimisation of beta-adrenergic receptor blocking agents by heart failure nurses in a hospital-based clinic to be a safe and efficient strategy [20]. They recruited 234 patients to attend a HF clinic staffed by a heart failure nurse and pharmacist. During the clinic visit beta-adrenergic receptor blocking agents, angiotensin converting enzyme inhibitors (ACEIs) and angiotension receptor blockers were up-titrated according to a hospital based protocol. At the end of their study, the percentage of patients being prescribed high dose beta-adrenergic receptor blocker increased from 18% to 57% without adversely affecting electrolytes and renal function [20].

Our pilot study is the first randomised controlled trial of a NLT clinic that specifically targets the titration of beta-adrenergic receptor blocking agents in an outpatient setting.

### Limitations

There were several limitations. The number of excluded patients was quite high. Many patients were already on optimal doses of beta-adrenergic receptor blocking agents. Secondly, the number of patients that declined to participate. There were a number of patients in employment or experienced difficulty in finding transport to the clinic. Unfortunately the clinic was only held on a Monday morning so there was no flexibility in terms of scheduling appointments around work commitments and transport availability. Scheduling of appointments needs to be more flexible, particularly in the day of the week that the clinic is available such as every second day or alternating morning with afternoon sessions.

Another limitation was that the pilot study was restricted to up-titration of beta-adrenergic receptor blocking agents and not other medications mandated in chronic HFrEF patients such as blockade of the renin-angiotensin-aldosterone system. In addition, the pilot study cohort only included patients that could read and speak English. Nevertheless, because of the more frequent personal contact rather than relying on instructions, one might potentially expect even greater benefit in patients from a non-English speaking background. A larger randomised trial would be necessary to assess clinical events.

## Conclusion

The pilot NLT clinic resulted in a rapid optimization of beta-adrenergic receptor blockers to target doses similar to those used in the large clinical CHF outcome trials. Given that mortality is reduced within only months of starting beta-adrenergic receptor blocking agents, consideration should be given to setting up these NLT clinics in parallel to usual heart failure clinics so as to facilitate rapid up-titration of these beneficial medications. These results suggest that a larger multi-centre cluster randomized controlled trial is warranted. Also consideration needs to be given to time and day of the clinic so it is also available to employed patients or those relying on relatives, who are usually working, for transport.
